# Criminal Mediation for Minors in Israel

**DOI:** 10.1100/tsw.2007.79

**Published:** 2007-03-02

**Authors:** Isack Kandel, Yael Arie, Sharon Glasner

**Affiliations:** ^1^ Faculty of Social Sciences, Department of Behavioral Sciences, Academic College of Judea and Samaria, Ariel, Israel; ^2^ Ministry of Treasury, Tel Aviv, Israel; ^3^ Public Defender Office, Ministry of Justice, Tel Aviv, Israel

**Keywords:** adolescence, delinquency, victim/offender, minors, prevention, Israel

## Abstract

Mediation was introduced in Canada in 1974 in order to handle a crime of robbery and vandalization committed by adolescents. After mediation, these adolescents agreed to apologize to each of their victims and pay restitution. Several countries (Canada, England, Finland, and the U.S.) have now made this opportunity available in the cases of young offenders. This review describes the process in the south of Israel. We find this method very powerful, but further studies are needed. Due to resource problems, it will not become mainstream in the near future.

## INTRODUCTION

Mediation is negotiation executed with the help of a third party. In the juvenile justice system, mediation took another approach in 1974, when two adolescents in Kitchener, Ontario, Canada, robbed and vandalized 21 homes. They paid for their crimes by visiting each of the 21 victims, apologizing for the damage they had caused, and paying restitution (called the Kitchener Experiment or the Victim/Offender Reconciliation Project, VORP)[1]. Rather than going through the full legal process, several countries (Canada, England, Finland, and the U.S.) have made this opportunity available in their juvenile justice systems, and young offenders meet their victims to discuss what the offender did and why, how the offense affected the victim, and how the offender might make amends. The parties have the full autonomy to control the procedure and decide for themselves what the desirable result is for them. In the mediation procedure, the parties themselves determine if an agreement will be achieved and how that agreement will look. In contrast to the judge or an arbiter, the mediator is powerless and cannot force the parties to any conflict resolution.

There are several basic objectives in the mediation procedure: the immediate objective of a current conflict resolution and the long-term objective in the relations between the parties. Change of the approach of the parties towards each other is also sought, so that each party will understand the point of view and the conception of the other regarding the subject of the conflict. This objective is particularly important for continuous relations, where the parties should continue and “live” with each other. The long-term objective is on the social comprehensive level, in order to create a new social qualification in the person, their self-image reinforcement, and an estimation of their ability to solve difficulties themselves without the need of professional and exterior factors.

Criminal mediation in its present form is typical of "retributive justice" (people should get what they deserve), but it also adopts "restorative justice", which encompasses a growing social movement to institutionalize peaceful approaches to harm, problem solving, and violations of legal and human rights. The mending justice school sees the offense as an insult to the victim, to society, and even to the delinquent himself. The goal of the mending justice is to try to cure the results of the offense, in contrast to the punitive justice school that persecutes the offender and disregards the victim. The main goal of the criminal mediation procedure is the repair of all parties involved. The delinquent should commit himself and take full responsibility whether to the victim or to the actions, while the victim should get the possibility to receive answers to the questions that bother him connected with the offense. The criminal mediation procedure is made in the presence of the delinquent, the victim, the representative of the community (depends on relation to the issues), and the mediator (a neutral body).

Each party is obviously interested in solving the conflict, which is the starting point of the entire procedure conducted according to the approach of Mark S. Umbreit from the School of Social Work at the University of Minnesota[2]. The following questions are asked during the criminal mediation procedure: what damage was caused? (and not which law was broken), to whom was the damage caused? (and not who did the damage), and who is responsible for damage repair? These questions urge the victim to repair the damage caused to him, and also place an emphasis on the repair and the return of the situation to its previous state rather than imposition of immediate punishment.

## EXPERIENCE FROM THE SOUTH OF ISRAEL

Since 1992, the Youth Probation Service in the south of Israel has worked with a criminal mediation program. Youth probation officers, qualified for this role through a special course, are responsible for this program. This program is offered to minors and their parents, as a repair intervention or as part of a more extensive treatment program. Results of the mediation procedure constitute the basis for the recommendations of the probation service to the police and the court. The professional training of the probation officer is within the framework of social work. The probation officer is responsible for the treatment of minor delinquents. He/she conducts an intervention with the goal to change the criminal behavior of the delinquent and to assist him to achieve positive change using a complex treatment program that includes mutual relations, that requires the creation of intervention suitable to each individual.

The treatment of delinquent minors in the Youth Probation Service is conducted according to two clauses in the Israeli Youth Law of 1971. Clause 22 is connected directly to the judicial procedure, referring to the role of the probation officer as an assistant of the court, and clause 12, which refers to the prevention of delinquency, safety of the minor, and rehabilitation. These instructions express the social nature of the law with a focus on assistance and rehabilitation.

## THE MINOR AND JUDICIAL POSITION

The role of education and development of minors is the foundation of family and society, with both having a vested interest in a healthy and productive growth. The legal age of adulthood in most countries is 18 years, but the legislature determines the rights and duties according to their interest and according to the variable age groups. The reaction of society to a crime committed by a minor is simultaneously adapted to his age. The younger the child, the more focus on treatment and rehabilitation, while by the time adulthood is reached, real punishment comes into play. Society also takes into account the severity of the crime, the criminal past of the minor, the need to deter the minor to commit more crimes in the future, and the danger to public safety.

## FROM THEORY TO PRACTICE

The complexity of the work with delinquent minors in general and the mediation in particular can be looked on from two angles. First, the mediation is with minors in the middle of a self-identity building process and, therefore, are busy testing boundaries. Second, they are in the middle of a criminal procedure, as a result of an offense they have committed, which threatens their reputation, their freedom, and their future. This feeling of fear stimulates mediation and most likely provides a development of responsibility and change in behavior. In order to facilitate such a process, the mediator must be equipped with knowledge, experience, and ability to deal with doubtfulness, fears, and lack of trust; to be neutral, emphatic, to listen; to be authoritative and consistent; and to respect the minor, even if the minor has committed a severe offense.

The Youth Probation Service is authorized by power to mediate, and when a violation has been committed by a minor, the details are transferred to the probation service who will decide if the specific case should be handled by the mediation procedure rather than a treatment procedure.

### First Stage (Early Preparation)

This stage is the initial stage, where the parties are not familiar with the procedure. Additionally, the parties come from different positions, and the victim often feels angry with the law and the justice system that led them to a situation without involvement in the criminal procedure. Therefore, it is very important to have separate meetings with each of the parties in order to build mutual confidence. The building of mutual confidence is important because both the victim and the offender will understand “that they are taken seriously”.

At these meetings, the parties receive a detailed explanation of the procedure, goals, and advantages. In case of a minor, the option of mediation is realized only after examination to determine if the minor is able and willing to participate in this procedure. The minor is required to admit the violation and his denial can disqualify him completely or delay his admission. If the minor is unable or not suitable, his record will be returned to the police for indictment.

An additional opportunity will arise at the court, where the mediation procedure will be offered to him again. At this stage, if there is an agreement of both parties, the mediation procedure can start.

### Second Stage (Agreement)

After the minor has received a letter, a telephone conversation, and, finally, the preparation meeting with his parents present, the minor has to accept or decline the mediation procedure. If accepted, the minor will be asked in detail about the event and his feelings about it. When the agreement to the mediation procedure is obtained, the hearing in court will be postponed until after the conclusion of the mediation procedure. At the end of the procedure, the judicial proceedings will continue and the results of the procedure will be presented to the court.

### Third Stage (Meeting between Victim and Offender)

Both the approach of Umbreit[2] and the transformation model introduced here are very important to open communication channels between the parties. First, each party will tell the “story of the conflict” from their point of view and express feelings about the event. During the procedure, it is not allowed to disturb or interrupt each other. The goal of the free conversation is “to release steam” and to enable the parties to tell about the offense, the damages caused to them, about their personal circumstances, and about the difficulties dealing with them.

The dialog between the victim and the offender is very important. The victim needs the recognition for the suffering endured. The victim also needs to know: why me? If the victim can see that the offender recognizes him as a person, becomes aware of the deed, shows remorse, and even expresses a desire to reimburse, then the victim will see that the system or the mediation will bring justice and does not ignore his needs.

The offender will need to take responsibility for his actions, to move from a passive level into an active level of reimbursement, where the mediation procedure has placed him. For the first time, the offender is forced to sit and listen to a victim made out of “flesh and blood” and not an image, without looking for excuses and justifications for the offense committed. He will reveal his story, the circumstances that led him to commit the offense, and he will express his desire for rehabilitation. During the mediation procedure, he will see that the system has given him a one-time opportunity to rehabilitate himself, which will show that “the system” is ready to trust him.

After each side has told his story, the mediator will facilitate a dialog that will examine the future implications, their desires, and the possibilities they have for a solution to the conflict.

### Fourth Stage (Formulation of the Agreement)

At this stage, there is an attempt to reach an agreement arranged by both sides. In the agreement, the damages caused to each party as a result of the act, the conclusions, and the future reimbursement will be specified.

### Fifth Stage (Presentation of the Agreement to the Court)

The agreement will serve as a document for the court in order to either close the record or to proceed with the treatment of the minor by the probation service in the framework of punishment.

## MEDIATION VS. PUNISHMENT

The purpose of punishment according to the classic model presents the utilitarian approach, which presumes that the purpose of the punishment is to deter and to prevent. It can be argued that the delinquent who agrees to the mediation procedure will not fear the results of the mediation and its implications, and therefore the deterrence obtained will be less and the risk for recidivism increased.

This point is directly connected to the purpose of prevention. The individual prevention point of view is the smaller the deterrence effect, the smaller the personal will of the delinquent to prevent the offense. On the other hand, it can be claimed that the obtained rehabilitation in the context of mediation is more effective prevention than the rehabilitation in the context of prison, but not every case (like severe violations) is suitable for the mediation procedure.

According to the rewarding approach, the mediation procedure is a compatible and proper procedure. This approach sees the offender as an autonomous person that takes responsibility for his actions. Therefore, he receives his penalty, either in the way of mediation or through the compensation he has to perform.

The first and the most necessary term to the opening of the procedure is that the offender confesses and intends to take responsibility for his actions, and the agreement of the victim to participate in the procedure. The consent of the victim to the procedure can lead to different punishments for the same crimes. The criminal that gets the consent of the victim will enter the procedure, while another offender with the same offense that does not get the consent of the victim is at a disadvantage in court. The different punishments imposed on the same crimes will have an influence on the offenders regarding responsibility for the actions, regarding the feeling of frustration, regarding the estrangement from society, and regarding disbelief in the system.

## TO MAKE AN EXAMPLE OR NOT

The central problem in this procedure is the absence of public awareness. Absence of publication hurts the social ability to learn from the event. In the mediation procedure, great emphasis is placed on the needs of the victim. The relation is to the victim that was hurt from the act and not to the law or the public in a direct way. The only participants in the mediation procedure are the victim, the offender, the mediator, and the community representative. If the mediation succeeded, the court will examine ways to mitigate the punishment of the offender and to close the record.

Compared with this, the punitive approach presumes that the committed offense is more than just damage caused to the victim; therefore, the involvement of the state is necessary in order to punish the delinquent. By the imposition of the penalty, society expresses denunciation and aversion of the deed. The denunciation and aversion will serve two purposes: teach the public that such actions receive reaction of the legal authority and bring the offender to take responsibility for his actions.

The mediation model presents a different philosophical point of view regarding crime and the system of justice. It represents the rehabilitative approach, but the procedure takes time and staff with a prolongation of the legal process.

## CONCLUSIONS

The criminal mediation procedure raises complicated value, moral, and judicial issues. These issues are complex and there are no clear guidelines for implementation, since each case is unique. We found that criminal mediation among minors is impressive. In the preliminary meeting between the offender and the victim, we observed that step by step, the parties began to relent and to discover each other, including feelings, emotions, and desires, regarding the past, the present, and the future.

The mediation procedure has received growing interest, but the absolute number of implemented files is relatively low due to the issue of resources.
